# Characterization of Small Molecule Inhibition of Avian Influenza Hemagglutinin

**DOI:** 10.1063/4.0001125

**Published:** 2025-10-27

**Authors:** Amir Shimon

**Affiliations:** UIC, Chicago, IL, USA

## Abstract

Influenza virus causes over 300,000 respiratory deaths per year. Moreover, the 1918 H1N1 influenza pandemic resulted in over 50 million deaths worldwide. Of particular concern are influenza strains H5N1 and H7N9 that currently circulate in avians and occasionally cross over to humans. In the case of humans infected with avian influenza, the mortality rate can rise to >50%. Influenza entry is mediated by the membrane protein hemagglutinin (HA). The Caffrey laboratory is interested in characterizing the HA mechanism of action and exploit this information for the design of novel antiviral therapeutics. In this poster we present the characterization of HA and its interactions by a combination of disciplines including virology, biochemistry, x-ray crystallography and Cryo EM. Specifically, potential inhibitors are discovered by screening libraries of small molecules using a virus-like-particle (VLP) system. Binding of the small molecule inhibitor is validated by NMR, Thermal Shift Assay (TSA), and Surface Plasmon Resonance (SPR). X-ray crystallography and Cryo EM are then used to give high resolution structural information of the binding site, thereby giving insight into the mechanism of drug inhibition and guidance for chemical optimization.

